# Supplementary material for: Cognitive Aging in Older Breast Cancer Survivors
Source: Cancers (Basel). 2023 Jun 16;15(12):3208. doi: 10.3390/cancers15123208 (PMC10296698; doi:10.3390/cancers15123208)
Supplement: Supplementary file 1 [file cancers-15-03208-s001.zip › cancers-2407925-supplementary.pdf]

Supplemental: Cognitive aging in older breast cancer survivors

James C. Root, Yuelin Li, Elizabeth Schofield, Irene Orlow, Elizabeth Ryan, Tiffany Traina, Sunita K. Patel and Tim A. Ahles

Summary This Supplemental provides a tutorial on the Bayesian "varying slopes and intercepts" model. It is intended to help readers who are unfamiliar with it to form a more intuitive understanding of the model. It is not intended to be an in-depth summary on Bayesian modeling. Several introductory textbooks provide a more complete coverage on the Bayesian approach to hierarchical data.[1,2] The book by Kruschke [3] is an accessible tutorial on the basics of Bayesian statistics. The "varying slopes and intercepts" concept is not new. Raudenbush and Bryk [4] was among the first to introduce social scientists to the idea of modeling varying slopes and intercepts in hierarchical data. Below we first illustrate what the "varying intercepts and slopes" are and what they represent in the context of this study. Next, we cover the model equations to introduce readers to the notation commonly used in Bayesian statistics.[1,2] We then describe selected model parameters in relation to this paper. The computer code, written in the statistical programming language R, can be found online (https://github.com/bayesnp/RandomSlopesIntcpts).

Illustrative examples of "varying intercepts and slopes"

Each study participant was assessed up to 4 times over 24 months. Supplement Figure S1 plots the repeated measures of observed APE scores for randomly selected survivors and controls. Plotted in blue are regression lines fitted to each participant's 4 assessments over time. For instance, one participant in the control cohort shows an intercept $\alpha = 0.7$ and a slope $\beta = 0.04$. The $\alpha = 0.7$ represents the person's APE score at month 0 (study enrollment), a standardized score of 0.7 above the norm in the first assessment occasion completed by this participant. This participant's slope of $\beta = 0.02$ represent her average change in APE score per month. Thus, the estimated score at 24 months is the intercept 0.7 plus the slope 0.02 multiplied by 24, which yields 1.18. On the right panel, we identify the intercept of -1.06 and slope of 0.01 for a participant in the survivor cohort. She scores 1.06 standard deviation below the norm at the first assessment occasion. These person-specific intercepts α_i and slopes β_i are what we refer to as the "varying intercepts and slopes".

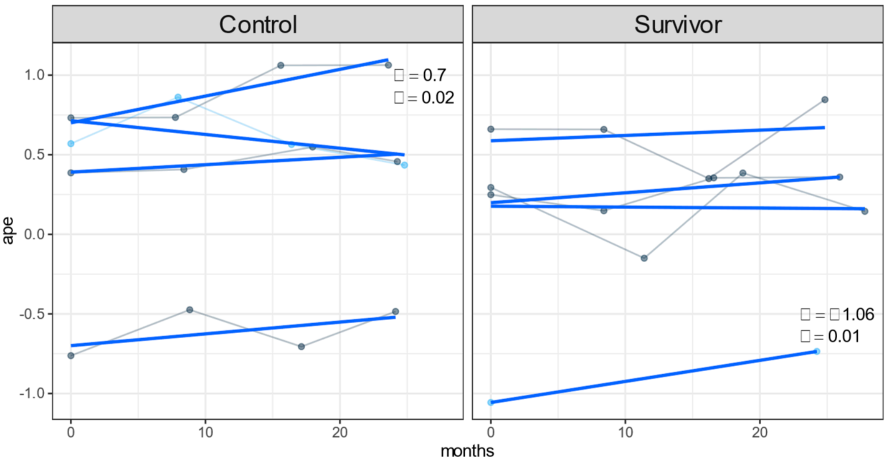

Supplement Figure S1. Examples of random intercepts and slopes fitted to longitudinal measurements of APE scores.

The person-specific intercepts represent each person's score at enrollment — the cognitive performance at the first assessment occasion. This first assessment is what Salthouse[5] characterizes as the *cross-sectional* score, while the repeated assessments the *longitudinal*

score. This terminology is in part attributed to a well-documented finding in aging research, that within-person cognitive changes are affected by repeated test exposure. The typical result is a practice effect that cognitive performance improves over repeated test administrations. The practice effect is visible above in the mostly slight upward slopes above. By contrast, the first assessment is less prone to practice effect, and presumed absent in participants who have never encountered the test before. To address this issue, the cross-sectional and longitudinal scores are often analyzed separately.[5] However, we argue that we do not have to analyze them separately. Our Bayesian approach is designed to fit all available data by leveraging the random intercepts and slopes.

Bayesian model equations

Consider the current study of n participants on cognitive performance $y_{i[t]}$ from the ith person at assessment time t, where the bracketed index $i[t]$ denotes that the assessments are nested within study participants. The Bayesian "varying intercepts and slopes" model is divided into two-levels, using the multi-level modeling approach in the literature.[4] In level 1, the repeated assessments for each person is first distilled into an intercept α_i and slope β_i:

$$
\begin{aligned}
&\text{Level 1:} \\
y_{i[t]} &\sim N\left(\alpha_i + \beta_i Months_{i[t]}, \sigma_\epsilon^2\right), \quad \text{for } i = 1, \ldots, n; t = 1, \ldots, 4 \\
&\text{Level 2:} \\
\begin{pmatrix} \alpha_i \\ \beta_i \end{pmatrix} &\sim N\left(\begin{pmatrix} \hat{\mu}_{\alpha_i} \\ \hat{\mu}_{\beta_i} \end{pmatrix}, \Omega\right) \\
&\text{where} \\
\hat{\mu}_{\alpha_i} &= \gamma_{00} + \gamma_{01} Age_i + \gamma_{02} Age_i^2 + \gamma_{03} survivor_i + \\
&\qquad \gamma_{04} survivor_i \cdot \mathrm{Age}_i + \gamma_{05} survivor_i \cdot Age_i^2 \\
\hat{\mu}_{\beta_i} &= \gamma_{10} + \gamma_{11} survivor_i + \gamma_{12} age4Q_i + \gamma_{13} survivor_i \cdot age4Q_i \\
\Omega &= \begin{pmatrix} \sigma_\alpha & 0 \\ 0 & \sigma_\beta \end{pmatrix} R \begin{pmatrix} \sigma_\alpha & 0 \\ 0 & \sigma_\beta \end{pmatrix} \\
R &\sim \mathrm{LKJCorr}(2) \\
\gamma_{..} &\sim N(0, 2.5) \\
\sigma_\epsilon^2 &\sim Exponential(1)
\end{aligned}
$$

The varying intercepts and varying slopes are further analyzed in level 2, where the intercepts α_i and slopes β_i are drawn from a bivariate normal distribution with averages $\begin{pmatrix} \hat{\mu}_{\alpha_i} \\ \hat{\mu}_{\beta_i} \end{pmatrix}$ and a 2x2 covariance matrix Ω. Recall that the intercept α_i represents the person's first assessment score (practice effect absent) and the slope β_i represents the within-person change over months (affected by practice effect). The model allows us to incorporate all available data, but in level 2 of the model we analyze the intercepts and slopes separately to avoid the confounding practice effect.

In level 2, $\hat{\mu}_{\alpha_i}$ represents the intercepts from 328 survivors and 158 controls. They are fitted as a quadratic function of age. For controls, their quadratic age function has the shape $\gamma_{00} + \gamma_{01} Age_i + \gamma_{02} Age_i^2$, where γ_{00} represents the overall intercept, γ_{01} the linear age term, and γ_{02} the quadratic age term. Survivors have a separate quadratic age function, and γ_{03}, γ_{04}, and γ_{05}, represent respectively the differences between survivors and controls in the overall intercept, the linear age term, and the quadratic age term. Note that, despite its complex notation, this equation is simply

a regression on the first assessment scores (practice effect absent) as a function of chronological age in quadratic form. Generally, in a quadratic curve, the intercept represents estimated score when the x-axis is at 0. In our model, we center age so that the overall intercept represents the estimated score at the average age of 72.5 for the entire sample. The linear term represents, generally, the overall growth over chronological age; and the quadratic term represents the curvature in the curve over chronological age. Figure S1 in the paper plots the chronological age trends in controls and survivors. The curve for controls is plotted using $\gamma_{00} + \gamma_{01} Age_i + \gamma_{02} Age_i^2$, where we plug in $\gamma_{00} = 0.013$, $\gamma_{01} = -0.047$, and $\gamma_{02} = 0.00001$ from Table 2.

Similarly, $\hat{\mu}_{\beta_i}$ represents the slopes from 328 survivors and 158 controls. They represent the estimated within-person change in cognition per month. They capture the practice effect over repeated assessments. They are modeled as a function of 4 age quartiles in the age4Q predictor. This effectively fits 8 separate longitudinal effects, 4 for each of the age quartiles in controls and 4 for survivors. It is possible to model age as a continuous predictor rather than categorical age quartiles because the model is flexible. However, we opt for age quartiles because they make the interpretation easier, and we can still examine within-person practice effects between the four age quartiles (60 to 67; 68 to 71; 72 to 76; and 77 to 90). For instance, in Figure S1, all age groups show practice effect over 24 moths except controls in the oldest age group (age 77 to 90).

The last 4 lines of the equation describe the priors in this application. We use the Bayesian approach in Sorensen, et al.[6] The covariance Ω for the varying intercepts and slopes is constructed by factoring it into separate standard deviations σ_α, σ_β and a correlation matrix R.[6] The correlation matrix R follows the LKJ prior, [7] which offers advantages over the inverse Wishart distribution for a covariance matrix. The LKJCorr(2) prior with a shape parameter of 2 represents a weak concentration of correlations between -0.5 and +0.5.[8] The remaining lines express the priors for the coefficients $\gamma_{..}$, as normal distributions centered at 0 with a 2.5 standard deviation for a stretched tail (https://cran.r-project.org/web/packages/rstanarm/vignettes/priors.html). The error standard deviation has a prior of exponential(1).

Finally, an added advantage of taking a Bayesian approach is that it yields useful information not available in conventional approaches. Supplement Figure S2 shows an example on how Bayesian computation estimates the age effect (γ_{01} for the control cohort).

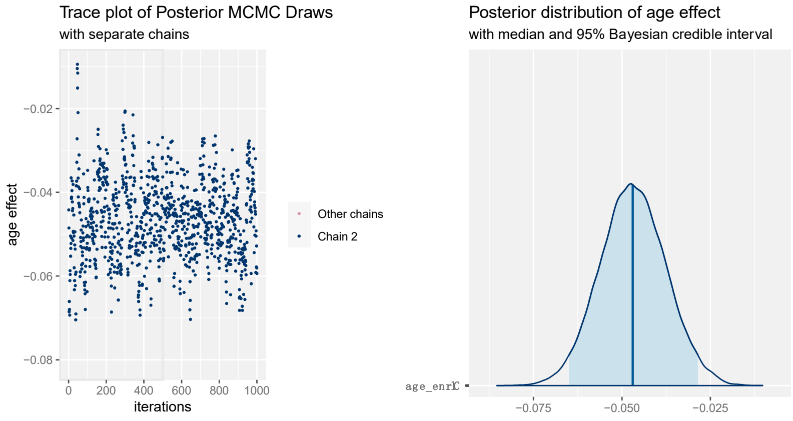

Supplement Figure S2. Example plots of Bayesian computation. The left plot shows that Bayesian computation draws random samples of age effect from the posterior distribution over 1,000 iterations. In this example, 4 independent sampling chains are drawn in parallel and combined in the right plot, which plots the model-estimated posterior density of the age effect. The shaded area represents the 95% Bayesian credible interval for the age effect.

To estimate the model coefficient, the Bayesian computer program draws sample from the posterior distribution of the γ_{01} coefficient over 1,000 iterations, one value at each iteration. In this example, 4 independent chains of 1,000 iterations each are drawn in parallel. The parallel chains help the user evaluate convergence between the chains. The user may begin with a default number (e.g., 2,000) and increase the number of iterations until a convergence criterion is reached. The samples are combined and plotted on the right to show the estimated posterior distribution of γ_{01}. The Bayesian 95% credible interval for the age effect is the interval that covers 95% of the simulated values.

The simulated samples can be used to calculate the chronological age gap between survivors and controls on the APE domain (Figure S1a in the paper, subplot on the right). The GitHub page for this paper provides the R syntax code to do the calculation. Basically, at each iteration, we get a set of coefficients from γ_{00} through γ_{05}. Next, we plug them into the model equation to generate one predicted value of $\hat{\mu}_{\alpha_i}$ for survivors or controls at a specific chronological age. We do this across the entire chronological age range to obtain the estimated average cognitive performance for survivors and controls. These are then plotted as the blue and red lines in Figure S1. When we repeat this step over 1,000 iterations, we get a distribution of 1,000 estimated values. The credible interval for this distribution of 1,000 numbers, when plotted over chronological age, gives us the shaded areas in Figure S1.

References

1 Gelman, A. & Hill, J. *Data analysis using regression and multilevel/hierarchical models*. (Cambridge University Press, 2007).

2 McElreath, R. *Statistical Rethinking: A Bayesian Course with Examples in R and Stan*. (CRC Press, 2016).

3 Kruschke, J. K. *Doing Bayesian Data Analysis, Second Edition: A Tutorial with R, JAGS, and Stan.*, (Academic Press / Elsevier, 2015).

4 Raudenbush, S. W. & Bryk, A. S. *Hierarchical Linear Models*. 2 edn, Vol. 1 (Sage Publications, Inc., 2002).

5 Salthouse, T. A. Trajectories of normal cognitive aging. *Psychol Aging* **34**, 17-24 (2019). https://doi.org:10.1037/pag0000288

6 Sorensen, T., Hohenstein, S. & Vasishth, S. Bayesian linear mixed models using Stan: A tutorial for psychologists, linguists, and cognitive scientists. *Quant Meth Psychol* **12**, 175-200 (2016). https://doi.org:10.20982/tqmp.12.3.p175

7 Lewandowski, D., Kurowicka, D. & Joe, H. Generating random correlation matrices based on vines and extended onion method. *J Multivariate Anal* **100**, 1989-2001 (2009). https://doi.org:10.1016/j.jmva.2009.04.008

8 Baldwin, S. *LKJ Priors,*, <https://psychstatistics.github.io/2014/12/27/d-lkj-priors/> (**2014, December 27**).
